# Gestational Exposure to Nonsteroidal Anti-Inflammatory Drugs and Risk of Chronic Kidney Disease in Childhood

**DOI:** 10.1001/jamapediatrics.2024.4409

**Published:** 2024-12-23

**Authors:** You-Lin Tain, Lung-Chih Li, Hsiao-Ching Kuo, Chiu-Ju Chen, Chien-Ning Hsu

**Affiliations:** 1 Department of Pediatrics, Kaohsiung Chang Gung Memorial Hospital and Chang Gung University College of Medicine, Kaohsiung, Taiwan; 2 Institute for Translational Research in Biomedicine, Kaohsiung Chang Gung Memorial Hospital and Chang Gung University College of Medicine, Kaohsiung, Taiwan; 3 Division of Nephrology, Kaohsiung Chang Gung Memorial Hospital and Chang Gung University College of Medicine, Kaohsiung, Taiwan; 4 Department of Pharmacy, Kaohsiung Chang Gung Memorial Hospital, Kaohsiung, Taiwan; 5 School of Pharmacy, Kaohsiung Medical University, Kaohsiung, Taiwan

## Abstract

**Importance:**

Gestational exposure to nonsteroidal anti-inflammatory drugs (NSAIDs) may increase the risk of adverse fetal kidney outcomes. However, details regarding timing, specific NSAIDs, and long-term childhood kidney outcomes are limited.

**Objective:**

To evaluate the association between gestational exposure to NSAIDs and the risk of chronic kidney disease (CKD) in childhood.

**Design, Setting, and Participants:**

This national cohort study assessed 1 025 255 children born alive in Taiwan from January 1, 2007, to December 31, 2017, with follow-up until December 31, 2021. Children without valid maternal-child linkage and with incomplete birth information were excluded. Data analysis was performed from November 30, 2023, to April 30, 2024.

**Exposure:**

Maternal prescriptions for NSAIDs from the last menstrual period to birth.

**Main Outcomes and Measures:**

The main outcome was childhood CKD, including congenital anomalies of the kidney and urinary tract and other kidney diseases. Cox proportional hazards regression models with stabilized inverse probability of treatment weighting (weighted hazard ratio [wHR]) and a robust sandwich estimator were used to estimate the relative risk of NSAID exposure in pregnancy, adjusted for newborn characteristics.

**Results:**

This study included 163 516 singleton-born children (24.0%) whose mothers (mean [SD] age at birth of child, 31.25 [4.92] years) used at least 1 dispensing of an NSAID during pregnancy. Gestational NSAID exposure was significantly associated with a higher risk of childhood CKD (wHR, 1.10; 95% CI, 1.05-1.15). No association was observed between NSAID use and fetal nephrotoxicity in sibling comparisons. Elevated risks were revealed for exposure during the second trimester (wHR, 1.19; 95% CI, 1.11-1.28) and the third trimester (wHR, 1.12; 95% CI, 1.03-1.22) in singleton-born children. Specific NSAID exposures associated with higher CKD risk included indomethacin (wHR, 1.69; 95% CI, 1.10-2.60) and ketorolac (wHR, 1.28; 95% CI, 1.01-1.62) in the first trimester, diclofenac (wHR, 1.27; 95% CI, 1.13-1.42) and mefenamic acid (wHR, 1.29; 95% CI, 1.15-1.46) in the second trimester, and ibuprofen (wHR, 1.34; 95% CI, 1.07-1.68) in the third trimester.

**Conclusions and Relevance:**

In this study, gestational exposure to NSAIDs was not associated with a substantial increase in the risk of childhood CKD when comparing between siblings. However, the findings underscore the need for caution when prescribing NSAIDs during pregnancy, particularly indomethacin and ketorolac in the first trimester, mefenamic acid and diclofenac in the second trimester, and ibuprofen in the third trimester, to ensure the safety of the offspring’s kidneys.

## Key Points

**Question:**

Is nonsteroidal anti-inflammatory drug (NSAID) use in pregnancy associated with an increased risk of chronic kidney disease (CKD) in offspring?

**Findings:**

This cohort study of 1 025 255 children in Taiwan found that gestational exposure to NSAIDs was associated with an increased risk of CKD in singleton-born children; however, a sibling comparison showed no association between gestational NSAID use and childhood CKD. A higher risk of CKD was associated with indomethacin and ketorolac exposure in the first trimester, diclofenac and mefenamic acid exposure in the second trimester, and ibuprofen exposure in the third trimester.

**Meaning:**

These findings suggest that NSAIDs should be used with caution during pregnancy, especially certain NSAIDs during specific trimesters.

## Teaser

This cohort study of children in Taiwan evaluates the association between NSAID exposure during different trimesters of pregnancy and the risk of chronic kidney disease in offspring during childhood.

## Introduction

Chronic kidney disease (CKD) is a global public health problem affecting more than 10% of the general population worldwide.^1^ Human nephron formation occurs at approximately weeks 5 to 36 of gestation.^2^ Exposure to an adverse maternal environment during fetal development may increase the risk of CKD later in life.^3,4,5^ Therefore, addressing the use of nephrotoxic medications during pregnancy is crucial for both maternal and fetal health.^6^

Nonsteroidal anti-inflammatory drugs (NSAIDs), such as indomethacin, inhibit prostaglandin synthesis and can reduce uterine contractions to delay preterm delivery.^7^ However, the US Food and Drug Administration recommends avoiding NSAIDs after 20 weeks of pregnancy due to the risk of oligohydramnios, kidney damage, and premature closure of the ductus arteriosus in the fetus.^8^ Cyclooxygenase (COX)-1 and COX-2 are key isoform enzymes in prostaglandin synthesis, and they have overlapping roles in physiologic functions.^9^ Currently, the safety of selectivity of COX inhibitors on long-term kidney outcomes in children exposed to NSAIDs during pregnancy remains uncertain.

Pregnant people commonly use NSAIDs as analgesics, with their use steadily increasing in Western countries from 1993 to 2013.^10^ Approximately 84% of pregnant people reported using over-the-counter NSAIDs during the first trimester.^11^ To address the knowledge regarding NSAID-related fetal nephrotoxicity, this study aimed to evaluate the association between NSAID exposure during different trimesters of pregnancy and the risk of CKD in childhood.

## Methods

### Study Design, Setting, and Population

This population-based cohort study of approximately 2 million maternal-child dyads used data from the Taiwan National Maternal and Child Health Database (MCHD) and Birth Reporting Database (BRD). An exact deterministic match between the mother and newborn was established using the mother’s identification number and delivery date in the BRD and MCHD between January 1, 2007, and December 31, 2017. The institutional review board of the Chang Gung Medical Foundation in Taipei, Taiwan, approved this study, and the requirement for informed consent was waived because of the retrospective design. All procedures were performed according to the guidelines of the Declaration of Helsinki.^12^ This study followed the Strengthening the Reporting of Observational Studies in Epidemiology (STROBE) reporting guideline.

**Figure 1.**
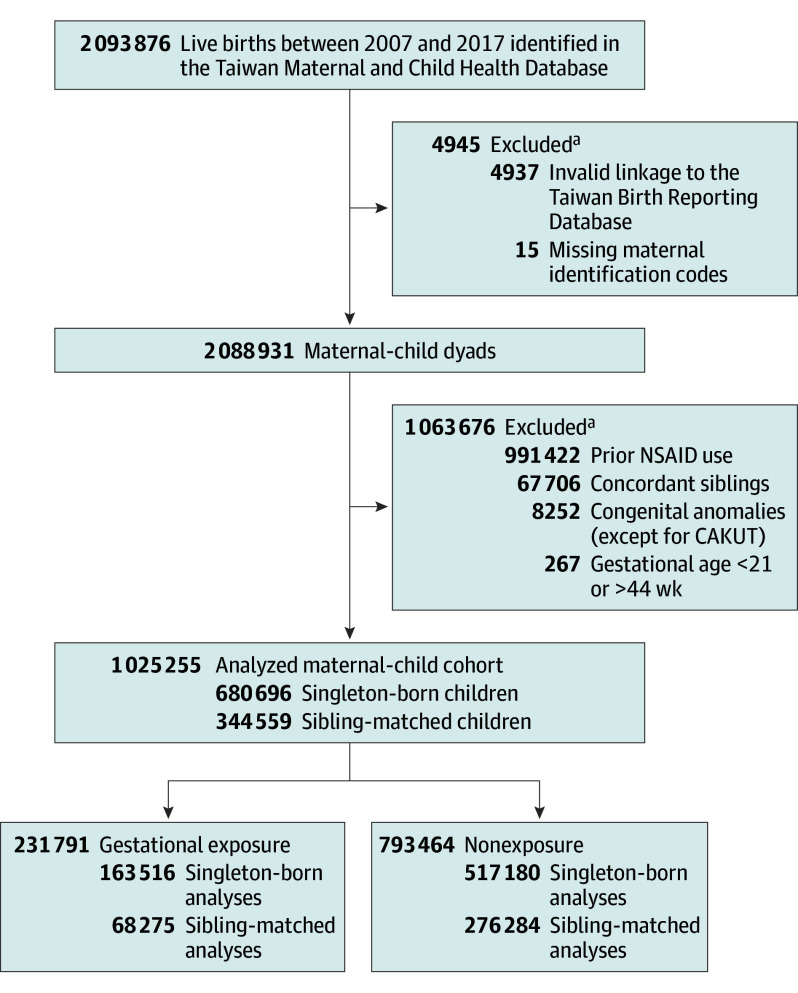
Flowchart of Maternal-Child Dyad Identification and Comparison Groups CAKUT indicates congenital anomalies of the kidney and urinary tract; NSAID, nonsteroidal anti-inflammatory drug. ^a^Some participants met more than 1 exclusion criteria.

Maternal-child pairs lacking valid linkages were excluded from the study. Medical records for follow-up were obtained from the Taiwan National Health Insurance Research Database (NHIRD) and death certificate database, covering the period from January 1, 2002, to December 31, 2021. This process resulted in an analytic sample of 2 088 931 maternal-child pairs, with 99.76% of the original sample retained (Figure 1). The characteristics of the Taiwan national health insurance program include universal, comprehensive health care services for more than 99% of the 23 million population and a national databank for all reimbursement-related data.^13^ A detailed description of the Taiwan NHIRD^14^ and mother-child dyads identified from the BRD and MCHD has been previously published, and diagnostic records, medication use, and use of health care services have been validated.^15,16,17^

Mothers without at least 180 days of medical information before the pregnancy were excluded to ensure solely gestational NSAID exposure (n = 0). Newborns with congenital anomalies were excluded, except for those with congenital anomalies of the kidney and urinary tract (CAKUT), to avoid confounding due to shared etiologic pathways for kidney disease. Concordant sibling pairs (n = 67 706) were excluded to create a discordant sibling-matched cohort because only discordant exposure and outcome status were informative for estimating effects.^18^ The final analytic cohort comprised 1 025 255 children, including singleton-born (n = 680 696) and discordant sibling-matched children (n = 344 559) (Figure 1).

### Maternal Use of NSAIDs in Pregnancy

The pregnancy period was defined as the time between the last menstrual period (LMP) and the delivery date. The LMP was estimated by subtracting the gestational age at birth from the delivery date. Additionally, the pregestational period was defined as any time before the LMP. The gestational period, comprising 3 trimesters (first, 0-90 days after the LMP; second, 91-180 days after the LMP; and third, 181 days until delivery), was defined as the period between the date of delivery minus the LMP.^19^

Exposure to NSAIDs, including aspirin and nonaspirin NSAIDs, was identified from prescription records, which included the medication name, date, number of pills, and duration of use throughout the pregnancy (eTable 1 in Supplement 1). Live newborns were categorized into 2 groups based on NSAID exposure during pregnancy: the nonexposure and gestational exposure groups. Infants whose mothers used NSAIDs during the pregestational period but discontinued or continued treatment during pregnancy were excluded to ensure internal validity (991 422 maternal-child dyads) (Figure 1). The intention-to-treat approach was applied to all mothers who had received at least 1 NSAID prescription.

### Offspring Outcomes

The outcome of interest was CKD incidence in children, defined by the date of the first inpatient or outpatient visits with a CKD diagnosis (eFigure 1 in Supplement 1) and used the *International Classification of Diseases, Ninth Revision* (*ICD-9*); *International Statistical Classification of Diseases*, *Tenth Revision* (*ICD-10*); *International Classification of Diseases, Ninth Revision, Clinical Modification* (*ICD-9-CM*); or *International Statistical Classification of Diseases, Tenth Revision, Clinical Modification* (*ICD-10-CM*) codes for CKD (eTable 2 in Supplement 1). According to the definition of CKD by Kidney Disease: Improving Global Outcomes,^20^ pediatric patients needed to have at least 1 *ICD-9* or *ICD-10* code for CKD as inpatients or at least 2 *ICD-9* or *ICD-10* codes for CKD within 1 year and at least 3 months apart as outpatients. The types of CKD were grouped as CAKUT and non-CAKUT. The condition was classified as primarily caused by CAKUT if the patients had *ICD-9* or *ICD-10* codes for both CAKUT and non-CAKUT, and the CKD event date was determined based on the CAKUT onset date. The follow-up time started from the date of birth until the event of CKD occurred or was censored owing to premature mortality, loss to follow-up, or the latest date in the database (December 31, 2021), whichever came first (eFigure 1 in Supplement 1).

### Covariate Assessment

Maternal characteristics, based on the most recent measurements before pregnancy, included a history of hypertension, hyperlipidemia, the Charlson Comorbidity Index,^21^ and the use of medications (based on prescription records) known to impact maternal health and study outcomes.^22^ These data were obtained from outpatient and inpatient settings before and during pregnancy (eTable 2 in Supplement 1). Offspring characteristics included sex, gestational age at birth, birth weight, and Apgar score at the first and fifth minutes (eTable 2 in Supplement 1).

### Statistical Analysis

Kaplan-Meier plots were created to estimate the cumulative incidence of CKD in children exposed and not exposed to gestational NSAIDs during the study period. The log-rank test was used to assess differences between the 2 groups.

The average treatment effect of gestational NSAIDs on CKD in children was estimated using the inverse probability of treatment weighting (IPTW) method to control for potential baseline confounding effects.^23^ Weights were assigned based on the inverse of the probability of receiving the treatment. The stabilized IPTW method was used to avoid bias from extreme weights and obtain a narrower CI.^24^ Stabilized weights were calculated by multiplying the patient’s original weight by the proportion of patients who received treatment. The probability of receiving vs not receiving NSAIDs during pregnancy was estimated using logistic regression with maternal characteristics (Table 1). Standardized weighted mean and proportion differences were calculated to assess the balance of baseline covariates between the exposed and nonexposed groups,^23^ and a difference of less than 0.1 was considered negligible.^25^

**Table 1.** Maternal Characteristics in the Singleton-Born Cohort Exposed and Nonexposed to Nonsteroidal Anti-Inflammatory Drugs^a^

Characteristic	No. (%) of mothers		Stabilized IPTW SMD
	Gestational exposure (n = 163 516)	Nonexposure (n = 517 180)	
Maternal age, y			
<25	18 198 (11.1)	43 055 (8.3)	0.0047
25-35	108 271 (66.2)	338 458 (65.4)	
36-44	36 801 (22.5)	134 640 (26.0)	
≥45	246 (0.2)	1027 (0.2)	
Medications during pregnancy			
Antidiabetes	1476 (0.9)	2932 (0.6)	0.0010
Systemic glucocorticoids	16 068 (9.8)	21 961 (4.2)	0.0088
Antihypertension	13 948 (8.5)	28 156 (5.4)	0.0117
Antiepileptics	325 (0.2)	607 (0.1)	0.0001
Antimicrobial agents^b^	89 580 (54.8)	136 566 (26.4)	0.0115
Statins	120 (0.1)	226 (0.04)	0.0022
Antidepressants	1493 (0.9)	2458 (0.5)	0.0085
Anticonvulsants	1015 (0.6)	1413 (0.3)	0.0012
Benzodiazepines	8299 (5.1)	10 284 (2.0)	0.0023
Immunosuppressive agents	42 (0.03)	57 (0.01)	0.0001
Prior medications (within 6 mo before LMP)			
Antidiabetes	962 (0.6)	2018 (0.4)	0.0020
Systemic glucocorticoids	13 561 (8.3)	32 203 (6.2)	0.0108
Antihypertension	692 (0.4)	1212 (0.2)	0.0028
Antiepileptics	363 (0.2)	788 (0.2)	0.0014
Antimicrobial agents^b^	39 656 (24.2)	99 149 (19.2)	0.0143
Statins	209 (0.1)	459 (0.09)	0.0007
Antidepressants	2254 (1.4)	4348 (0.8)	0.0048
Anticonvulsants	1135 (0.7)	2257 (0.4)	0.0051
Benzodiazepines	8923 (5.5)	18 816 (3.6)	0.0093
Immunosuppressive agents	38 (0.02)	64 (0.01)	0.0013
Gestational comorbidity			
Gestational diabetes	7482 (4.6)	23 357 (4.5)	0.0008
Gestational hypertension	1272 (0.8)	2125 (0.4)	0.0035
Preeclampsia	1301 (0.8)	970 (0.2)	0.0077
Anemia	914 (0.6)	1545 (0.3)	0.0015
Other maternal complications	132 665 (81.1)	365 331 (70.6)	0.0312
Prior comorbidity (within 6 mo before LMP)			
Acute myocardial infarction	3 (0.0)	6 (0.0)	0.0006
Congestive heart failure	117 (0.07)	250 (0.05)	0.0001
Peripheral vascular diseases	32 (0.02)	58 (0.01)	0.0003
Cerebral vascular accident	80 (0.05)	135 (0.03)	0.0004
Pulmonary disease	2529 (1.6)	4456 (0.9)	0.0040
Connective tissue disorder	533 (0.3)	853 (0.2)	0.0027
Peptic ulcer	1434 (0.9)	2786 (0.5)	0.0020
Liver diseases	922 (0.6)	2518 (0.5)	0.0022
Diabetes	3491 (2.1)	10 839 (2.1)	0.0016
Diabetes complications	103 (0.06)	179 (0.03)	0.0001
Paraplegia	15 (0.01)	22 (0.0)	0.0006
Kidney disease	121 (0.07)	253 (0.05)	0.0006
Cancer	428 (0.3)	1155 (0.2)	0.0006
Severe liver diseases	8 (0.0)	14 (0.0)	0.0006
Metastatic cancer	16 (0.01)	21 (0.0)	0.0003
Hypertension	2597 (1.6)	3715 (0.7)	0.0003
Hyperlipidemia	614 (0.4)	1454 (0.3)	0.0013

Abbreviations: IPTW, inverse probability of treatment weighting; LMP, last menstrual period; SMD, standardized mean difference.

^a^ Propensity score calculation included 38 maternal-related variables and all SMDs less than 0.1 in Table 1, indicating that the distribution of these variables is insignificant between comparison groups.

^b^ Aminoglycoside uses between gestational exposure and nonexposure were 2446 (1.5%) and 1220 (0.2%) during pregnancy and 581 (0.4%) and 1365 (0.3%) in the prior medications category.

For the singleton-born children cohort (primary analysis), Cox proportional hazards regression models with stabilized IPTW and a robust sandwich estimator were used to estimate conditional hazard ratios (HRs) and 95% CIs for newborn characteristics to examine the association between gestational NSAID exposure (vs nonexposure) and childhood CKD.^26^ To examine the consistency of the average treatment effect of NSAIDs, Cox proportional hazards regression stratified by quintiles of the propensity score (5 strata) was applied in the primary analyses.^26^

Several secondary analyses were conducted to explore the heterogeneity of NSAIDs and the robustness of the primary analysis results. First, to examine the risk window of NSAID exposure, the stabilized IPTW model in the singleton-born maternal-child cohort was stratified according to the gestational timing of NSAID exposure (first, second, and third trimesters). Second, because NSAIDs are recommended for treating premature closure of the patent ductus arteriosus in preterm infants, this aspect was also considered to avoid misclassification in the analyses.^27^ The exposure was redefined as an additional 60 days after delivery (gestational period plus 60 days) to ensure comprehensive capture of NSAID exposure. The IPTW was reassessed to estimate the average treatment effect of gestational NSAIDs in the same singleton pregnancy cohort. Third, stratified analyses by offspring’s sex, gestational age, and birth weight were conducted due to a higher CKD risk in boys and infants with intrauterine growth restriction or preterm birth.^28^ Fourth, to evaluate the risk heterogeneity of gestational NSAIDs concerning specific NSAID treatment during pregnancy, we repeated the primary analysis for aspirin, indomethacin, and 5 common NSAIDs (diclofenac, mefenamic acid, ibuprofen, ketorolac, and indomethacin) to assess their relative risk for CKD in childhood (eTable 1 in Supplement 1).

We conducted sibling-matched analyses for gestational NSAIDs to control for shared genetic and environmental confounding factors. We used stratified Cox proportional hazards regression and a robust sandwich estimator with a separate stratum for each family by the mother’s unique identification number. Statistical significance was set at a 2-sided *P* < .05 for all statistical analyses. SAS, version 9.4 (SAS Institute Inc) was used for data processing and analysis. Data analysis was performed from November 30, 2023, to April 30, 2024.

## Results

### Characteristics of the Study Cohort

Among the 680 696 singleton-born children analyzed, 163 516 (24.0%) had mothers who had at least 1 dispensing of an NSAID during pregnancy. Within this group with gestational NSAID exposure, the mean (SD) maternal age at child’s birth was 31.25 (4.92) years, with 66.2% of mothers being between 25 and 35 years of age. After weighting by IPTW, all measured maternal characteristics were similar between the exposed and nonexposed groups (standardized mean difference <0.1) (Table 1). The distributions of the logit of the propensity score of study participants between gestational exposure and nonexposure groups are shown in eFigure 2 in Supplement 1. In the overall cohort of 680 696 children, 52.5% were male and 47.5% were female, 7.1% were born before 37 weeks of gestation, and 6.7% had low birth weight (<2.5 kg) (eTable 3 in the Supplement).

### Patterns of NSAID Use During Pregnancy

Among 163 516 participants who used NSAIDs during pregnancy, 99 846 (61.1%), 54 034 (33.0%), and 43 447 (26.6%) used NSAIDs in the first, second, and third trimesters, respectively (eTable 4 in Supplement 1). Additionally, 124 558 participants (76.2%) used NSAIDs in early pregnancy (≤20 weeks of gestation).

Among 157 025 nonaspirin NSAID users, most received diclofenac (67 335 [42.9%]), primarily during their first and second trimesters, followed by mefenamic acid (55 828 [35.6%]) and ibuprofen (31 993 [20.4%]). Among all NSAID users, aspirin was used during pregnancy by 9032 participants (5.5%), and indomethacin was used by 7358 (4.5%); both drugs were mainly used during the second and third trimesters (eTables 1 and 4 in Supplement 1).

### Association Between Gestational NSAIDs and Offspring Kidney Outcomes

The median follow-up was 9.75 years (range, 0-15 years). A total of 10 547 children (1.6%) in the singleton-born cohort had CKD, including 7144 (1.0%) with non-CAKUT CKD and 273 (0.04%) requiring kidney replacement therapy (Table 2; eTable 5 in Supplement 1). The overall attributable risk of CKD was 1.79 per 1000 person-years and 1.61 per 1000 person-years in the gestational and nonexposure to NSAIDs groups, respectively (eTable 5 in Supplement 1). The all-cause mortality rate was higher in the gestational NSAID–exposed group compared with those in the nonexposed group (0.46 and 0.37 per 1000 person-years, respectively) (eTable 5 in Supplement 1). The cumulative incidence of CKD, CAKUT, and non-CAKUT during the study period was higher in the NSAID-exposed group compared with the nonexposed group (eFigure 3 in Supplement 1).

**Table 2.** Association Between Childhood Chronic Kidney Disease and Nonsteroidal Anti-Inflammatory Drugs in the Singleton-Born Child Cohort

Characteristic	No. of children	No. (%) of children with event	Stabilized IPTW Cox proportional hazards regression model^a^		Stratified PS Cox proportional hazards regression model^a^	
			wHR (95% CI)	*P* value	sHR (95% CI)	*P* value
Primary analysis	680 696	10 547 (1.6)	1.10 (1.05-1.15)	<.001	1.08 (1.04-1.14)	<.001
CAKUT	680 696	4981 (0.7)	1.08 (1.02-1.16)	.02	NA^b^	NA
Non-CAKUT	680 696	7144 (1.0)	1.12 (1.06-1.19)	<.001	NA^b^	NA
Exposure timing during pregnancy						
First trimester (LMP to <13 wk)	99 846	1667 (1.7)	1.07 (1.01-1.13)	.03	1.06 (1.00-1.12)	.048
Second trimester (13-26 wk)	54 034	1006 (1.9)	1.19 (1.11-1.28)	<.001	1.17 (1.09-1.25)	<.001
Third trimester (≥27 wk to delivery)	43 447	762 (1.8)	1.12 (1.03-1.22)	.008	1.10 (1.02-1.19)	.02
≤20 wk Gestation	124 558	2119 (1.7)	1.09 (1.04-1.15)	.001	1.08 (1.03-1.14)	.003
>20 wk Gestation	61 403	1127 (1.8)	1.17 (1.09-1.25)	<.001	1.15 (1.08-1.22)	<.001
Sex						
Male	357 062	5905 (1.6)	1.05 (0.98-1.12)	.16	1.03 (0.97-1.09)	.33
Female	323 634	4642 (1.4)	1.17 (1.09-1.25)	<.001	1.16 (1.08-1.23)	<.001
Birth weight, g						
<2500	45 308	774 (1.7)	1.04 (0.88-1.24)	.63	1.05 (0.90-1.22)	.56
≥2500	635 388	9773 (1.5)	1.10 (1.05-1.16)	<.001	1.09 (1.04-1.14)	.001
Gestational age, wk						
<37	48 472	867 (1.8)	1.09 (0.92-1.28)	.32	0.99 (0.86-1.15)	.92
≥37	632 224	9680 (1.5)	1.10 (1.05-1.15)	<.001	1.09 (1.04-1.15)	<.001

Abbreviations: CAKUT, congenital anomalies of the kidney and urinary tract; IPTW, inverse probability of treatment weighting; LMP, last menstrual period; PS, propensity score; sHR, stratified hazard ratio (hazard ratio derived from the stratified PS Cox proportional hazards regression model [5 strata]); wHR, weighted hazard ratio (hazard ratio derived from the IPTW Cox proportional hazards regression model).

^a^ Both the sHR and wHR models were adjusted with the same offspring characteristics using a robust sandwich estimator in the singleton-born cohort (n = 680 696).

^b^ sHR model was only performed in the primary analysis.

Gestational NSAID exposure was associated with an increased risk of CKD in childhood (weighted HR [wHR], 1.10; 95% CI, 1.05-1.15) conditional on fetal characteristics (eTable 3 in Supplement 1), and the estimated association was consistent with the propensity score stratification model (stratified HR, 1.08; 95% CI, 1.04-1.14) (Table 2). An increased risk of all-cause mortality was observed with gestational NSAID exposure after adjustment for fetal characteristics (wHR, 1.12; 95% CI, 1.03-1.23) (eTable 5 in Supplement 1). In stratified analyses, the association between NSAID exposure and CKD showed similar trends across different subgroups of gestational age (<37 and ≥37 weeks), birth weight (<2.5 kg and ≥2.5 kg), and sex (male and female), and the point-of-risk estimate was slightly higher for girls (wHR, 1.17; 95% CI, 1.09-1.25) (Table 2).

### Comparisons in Different Risk Window Exposure to NSAIDs

A significant association was found with increased risk estimates in the second trimester (wHR, 1.19; 95% CI, 1.11-1.28) and the third trimester (wHR, 1.12; 95% CI, 1.03-1.22). The estimated risk was higher for late exposure (≥20 weeks) (wHR, 1.17; 95% CI, 1.09-1.25) compared with early exposure (<20 weeks) (wHR, 1.09; 95% CI, 1.04-1.15) (Table 2). In models considering an additional 60-day exposure period after delivery, the exposure group included 521 577 NSAID users, and the risk estimate for CKD in childhood was close to null (wHR, 1.01; 95% CI, 0.96-1.06) (eTable 6 in Supplement 1).

### Comparisons in Gestational Exposure to Different NSAIDs

**Figure 2.**
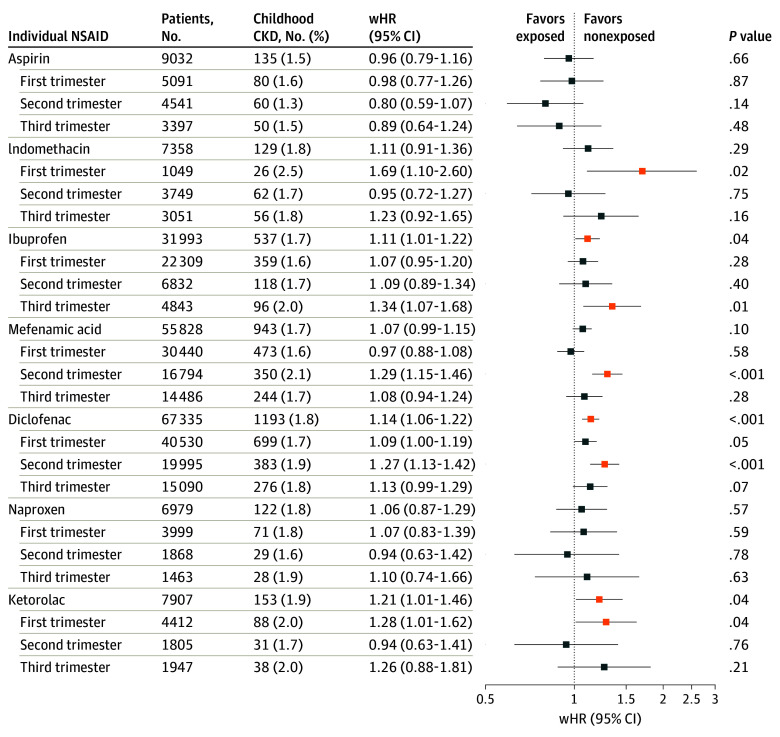
Association Between Childhood Chronic Kidney Disease (CKD) and Gestational Exposure to Nonsteroidal Anti-Inflammatory Drugs (NSAIDs) in the Common Uses of Individual NSAID Analyses wHR indicates weighted hazard ratio (the conditional hazard ratio derived from the stabilized inverse probability of the treatment-weighted Cox proportional hazards regression model adjusted with offspring characteristics using a robust sandwich estimator in the singleton-born cohort of 680 696 children).

The absolute rates of childhood CKD varied among aspirin, indomethacin, and 5 commonly used NSAIDs in pregnancy (Figure 2; eTable 1 in Supplement 1), and the wHR of CKD significantly increased with gestational exposure to ibuprofen (wHR, 1.11; 95% CI, 1.01-1.22), diclofenac (wHR, 1.14; 95% CI, 1.06-1.22), and ketorolac (wHR, 1.21; 95% CI, 1.01-1.46) (Figure 2).

The risk estimates were notably higher for specific NSAIDs during different trimesters: indomethacin (wHR, 1.69; 95% CI, 1.10-2.60) and ketorolac (wHR, 1.28; 95% CI, 1.01-1.62) in the first trimester, mefenamic acid (wHR, 1.29; 95% CI, 1.15-1.46) and diclofenac (wHR, 1.27; 95% CI, 1.13-1.42) in the second trimester, and ibuprofen (wHR, 1.34; 95% CI, 1.07-1.68) in the third trimester (Figure 2). Little variation in relative risk measures at different risk window exposures was observed for aspirin and naproxen (Figure 2). No dose-response relationship between aspirin or naproxen exposure and CKD risk in childhood was found in the study cohort (eTable 7 in Supplement 1).

### Sibling-Matched Children Cohort Analyses

In the sibling-matched pairs with discordant exposure to NSAIDs (n = 344 559), there was no significant difference in sex between the NSAID-exposed and nonexposed groups (eTable 8 in Supplement 1). The overall attributable risk of non-CAKUT CKD was higher in the NSAID-exposed group (1.67 per 1000 person-years) than in the nonexposed group (1.50 per 1000 person-years) (eTable 9 in Supplement 1). Adjusting for fetal characteristics, sibling-matched analyses showed no statistical association (wHR, 1.05; 95% CI, 0.97-1.13) between gestational NSAID use and childhood CKD (eTable 10 in Supplement 1). The effect estimates of NSAID exposure on CKD in childhood were consistent across different risk windows and fetal characteristics (eTable 10 in Supplement 1).

## Discussion

This study found that many newborns were exposed to NSAIDs during the second or third trimester. Such exposure was associated with a moderate increase in risk of childhood CKD, varying by NSAID and exposure timing. Although sibling comparisons were underpowered, the number needed to harm ranged from 476 to 556 for CKD among singleton-born and sibling-matched children. Exploratory analyses support avoiding potential nephrotoxic NSAID exposure during pregnancy due to its association with childhood CKD.^8^

To our knowledge, this is the first population-based cohort study in 15 years to investigate the association of gestational exposure to NSAIDs and childhood CKD. Although the association was significant, the magnitude of the risk estimate was relatively small considering the nature of a large population observational study. A 12% to 19% increased risk of CKD with later pregnancy exposure to NSAIDs highlighted the importance of timing in relation to the nephron development process.^29^

NSAID nephrotoxicity in the fetus is related to the inhibition of prostaglandin synthesis, as NSAIDs can cross the placenta and impair fetal kidney development (eTable 11 in Supplement 1).^3^ It may also be linked to maternal kidney damage during pregnancy.^30^ The labels for the warning and kidney safety of NSAIDs are given in eTable 11 in Supplement 1. NSAIDs have been extensively studied for fetal safety, particularly regarding late gestational exposure.^31^ For instance, nephrotoxicity of ibuprofen used in late gestation included reduced urine production in the fetus (oligohydramnios)^32,33^ and acute kidney injury (anuria) in neonates exposed to indomethacin less than 7 days before delivery.^34^

This study observed an increased risk of CKD in children whose mothers used indomethacin or ketorolac during the first trimester. These results suggest that first-trimester exposure to NSAIDs may carry the same risk of nephrotoxicity as later gestation exposure in childhood, potentially affecting nephrogenesis. The use of other common NSAIDs, including mefenamic acid, diclofenac, and ibuprofen, during later trimesters also increased the risk of CKD in childhood, with varying degrees of risk. Additionally, because most NSAIDs in Taiwan are available only by prescription, the likelihood of exposure misclassification due to over-the-counter availability (limited to ibuprofen) is minimal in this study.

The potential modification effect of neonatal characteristics on NSAIDs associated with childhood CKD diagnosis was higher in female infants than in male infants. A previous animal study indicated that NSAIDs may have lower efficacy and higher metabolism in female animals.^35^ Whether such sex differences extend to prostaglandin signaling during kidney development remains uncertain. The current study, however, did not observe any modification effect of low birth weight or prematurity on the association between NSAID exposure and the risk of CKD later in life.

### Limitations

This study has several limitations. First, exposure was based solely on prescription records, potentially leaning to exposure misclassification if actual NSAID use differed. Second, maternal factors, such as malnutrition and micronutrient deficiencies (eg, vitamin A),^36,37^ and behaviors, such as smoking^35^ and alcohol consumption,^36^ could influence kidney outcome. Although the sibling comparison is recommended to control for unmeasured, shared confounding factors, the design could attenuate the association and amplify confounding from nonshared factors.^18^ These study results underscore the need for larger cohort studies to address a specific residual confounding factor. Third, the study’s findings on NSAID use and outcome detection may not be generalizable to populations with health insurance systems. Fourth, CKD was based on diagnostic codes rather than laboratory measurements of glomerular filtration rate, possibly leading to incomplete or misclassified diagnoses of less severe CKD.

## Conclusions

In this cohort study, exposure to NSAIDs during pregnancy was associated with an increased risk of CKD in offspring, but a null association was revealed in sibling comparison. Findings suggest that caution should be taken when using indomethacin and ketorolac in the first trimester, mefenamic acid and diclofenac in the second trimester, and ibuprofen in the third trimester to ensure the safety of the child’s kidneys. These NSAIDs should be prescribed only after a thorough assessment of benefits and risks for both mother and child. Future research should investigate the specific roles of genetic and environmental factors in kidney development across different stages of pregnancy.

## Notes

Supplement. **eTable 1.** Uses of NSAIDs and cumulative incidence of CKD in the singleton-born child cohort (n = 163 516) **eTable 2.** Codes for medications and diagnoses used in the study **eTable 3.** Neonatal characteristics between comparison groups in the singleton-born child cohort (n = 680 696) **eTable 4.** Uses of NSAIDs by trimester in pregnancy in the singleton-born child cohort (n = 163 516) **eTable 5.** Incidence rate of study outcome in the singleton-born child cohort (n = 680 696) **eTable 6.** In sensitivity analyses, study outcomes in the additional 60-day exposure to NSAIDs period after the date of birth (n = 680 696) **eTable 7.** Dose-response between individual NSAID and risk of CKD in the singleton-born child cohort **eTable 8.** Neonatal characteristics in the sibling-matched child cohort (n = 344 559) **eTable 9.** Incidence rate of study outcome in the sibling-matched child cohort (n = 344 559) **eTable 10.** Associations of study outcomes with gestational exposure to NSAIDs in the sibling-matched child cohort analyses (n = 344 559) **eTable 11.** Descriptions of the common NSAIDs used in the study **eFigure 1.** Study design, exposure, and outcome time frame **eFigure 2.** The distributions of logit of the propensity score of subjects between gestational exposure and non-exposure to NSAIDs groups **eFigure 3.** Cumulative incidence of CKD in offspring over 15 years’ follow-up

Supplement 2.
Data Sharing Statement
