# Supplementary material for: Gestational Exposure to Nonsteroidal Anti-Inflammatory Drugs and Risk of Chronic Kidney Disease in Childhood
Source: JAMA Pediatr. 2024 Dec 23;179(2):171–8. doi: 10.1001/jamapediatrics.2024.4409 (PMC11791701; doi:10.1001/jamapediatrics.2024.4409)
Supplement: Supplement. — eTable 1. Uses of NSAIDs and cumulative incidence of CKD in the singleton-born child cohort (n = 163 516) eTable 2. Codes for medications and diagnoses used in the study eTable 3. Neonatal characteristics between comparison groups in the singleton-born child cohort (n = 680 696) eTable 4. Uses of NSAIDs by trimester in pregnancy in the singleton-born child cohort (n = 163 516) eTable 5. Incidence rate of study outcome in the singleton-born child cohort (n = 680 696) eTable 6. In sensitivity analyses, study outcomes in the additional 60-day exposure to NSAIDs period after the date of birth (n = 680 696) eTable 7. Dose-response between individual NSAID and risk of CKD in the singleton-born child cohort eTable 8. Neonatal characteristics in the sibling-matched child cohort (n = 344 559) eTable 9. Incidence rate of study outcome in the sibling-matched child cohort (n = 344 559) eTable 10. Associations of study outcomes with gestational exposure to NSAIDs in the sibling-matched child cohort analyses (n = 344 559) eTable 11. Descriptions of the common NSAIDs used in the study eFigure 1. Study design, exposure, and outcome time frame eFigure 2. The distributions of logit of the propensity score of subjects between gestational exposure and non-exposure to NSAIDs groups eFigure 3. Cumulative incidence of CKD in offspring over 15 years’ follow-up [file jamapediatr-e244409-s001.pdf]

Supplementary Online Content

Tain Y-L, Li L-C, Kuo H-C, Chen C-J, Hsu C-N. Gestational exposure to nonsteroidal anti-inflammatory drugs and risk of chronic kidney disease in childhood. *JAMA Pediatr.* Published online December 23, 2024. doi:10.1001/jamapediatrics.2024.4409

eTable 1. Uses of NSAIDs and cumulative incidence of CKD in the singleton-born child cohort (n=163,516)

eTable 2. Codes for medications and diagnoses used in the study

eTable 3. Neonatal characteristics between comparison groups in the singleton-born child cohort (n=680,696)

eTable 4. Uses of NSAIDs by trimester in pregnancy in the singleton-born child cohort (n=163,516)

eTable 5. Incidence rate of study outcome in the singleton-born child cohort (n=680,696)

eTable 6. In sensitivity analyses, study outcomes in the additional 60-day exposure to NSAIDs period after the date of birth (n=680,696)

eTable 7. Dose response between individual NSAID and risk of CKD in the singleton-born child cohort

eTable 8. Neonatal characteristics in the sibling-matched child cohort (n=344,559)

eTable 9. Incidence rate of study outcome in the sibling-matched child cohort (n=344,559)

eTable 10. Associations of study outcomes with gestational exposure to NSAIDs in the sibling-matched child cohort analyses (n=344,559)

eTable 11. Descriptions of the common NSAIDs used in the study

eFigure 1. Study design, exposure and outcome time frame

eFigure 2. The distributions of logit of the propensity score of subjects between gestational exposure and non-exposure to NSAIDs groups

eFigure 3. Cumulative incidence of CKD in offspring over 15 years' follow-up

This supplementary material has been provided by the authors to give readers additional information about their work.

© 2024 American Medical Association. All rights reserved.

eTable 1. Uses of NSAIDs and cumulative incidence of CKD in the singleton-born child cohort (n=163,516)

Exposure	ATC	Children exposed		Children with CKD	
		n (%)		n(%)	
**NSAIDs**		163516		2792	(1.71)
Aspirin (ASA)	B01AC06	9032	(5.52)	135	(1.49)
Non-ASA NSAIDs	M01AA, M01AB, M01AC, M01AE, M01AG, M01AH, M01AX (excld M01AX05)	157025	(96.03)	2696	(1.72)
**Lower COX- 2 selectivity**		60896	(37.24)	1042	(1.71)
Indomethacin	M01AB01, M01AB51	7358	(4.50)	129	(1.75)
Ibuprofen	M01AE01	31993	(19.57)	537	(1.68)
Naproxen	M01AE02	6979	(4.27)	122	(1.75)
Acemetacin	M01AB11	2319	(1.42)	36	(1.55)
Ketorolac	M01AB15	7907	(4.84)	153	(1.93)
Aceclofenac	M01AB16	901	(0.55)	15	(1.66)
Tolmetin (sodium)	M01AB03	42	(0.03)	0	
Piroxicam	M01AC01	2094	(1.28)	38	(1.81)
Tenoxicam	M01AC02	494	(0.30)	13	(2.63)
Ketoprofen	M01AE03	4033	(2.47)	64	(1.59)
Fenoprofen (calcium)	M01AE04	49	(0.03)	0	
Meclofenamic acid	M01AG04	1220	(0.75)	26	(2.13)
Nabumetone	M01AX01	62	(0.04)	≦3	NA
**Higher COX-2 selectivity**		114859	(70.24)	1976	(1.72)
Diclofenac	M01AB05	67335	(41.18)	1193	(1.77)
Alclofenac	M01AB06	8	<0.01.	0	
Meloxicam	M01AC06	504	(0.31)	7	(1.39)
Mefenamic acid	M01AG01	55828	(34.14)	943	(1.69)
Tolfenamic acid	M01AG02	68	(0.04)	≦3	NA
Flufenamic acid	M01AG03	155	(0.09)	≦3	NA
Celecoxib	M01AH01	72	(0.04)	0	
Etoricoxib	M01AH05	52	(0.03)	≦3	NA
Niflumic acid	M01AX02	22	(0.01)	≦3	NA
Nimesulide	M01AX17	299	(0.18)	≦3	NA
Etodolac micronized	M01AB08	471	(0.29)	8	(1.70)
Fenbufen	M01AE05	366	(0.22)	8	(2.19)
**Uncertain**		1245	(0.76)	31	(2.49)
Benzydamine HCL	M01AX07	241	(0.15)	5	(2.07)
Mepirizole	M01AX91	1008	(0.62)	26	(2.58)

1. COX-2 (cyclooxygenase-2) selectivity classification for the included NSAIDs (non-steroids anti-inflammatory drugs;) was based on relative COX selectivity (COX-2 versus COX-1) summarized recommendations [1]. ATC (Anatomy of Therapeutic Classification code)
3. NA: Number for event rate ≤3 is not shown due to the data security requirement.

References

[1] Bonnesen K and Schmid M. Recategorization of non-aspirin nonsteroidal anti-inflammatory drugs according to clinical relevance: Abandoning the traditional NSAID terminology. Canadian Journal of Cardiology 2021; 37 (11): 1705-1707.

© 2024 American Medical Association. All rights reserved.

eTable 2. Codes for medications and diagnoses used in the study

Variable	Code	Timing and Definition
**Chronic kidney disease diagnoses in childhood [1, 2]**		≥1 ICD-9/10 codes in inpatient setting or ≥2 ICD-9/10 codes in outpatient setting apart ≥90 days and <365 days, whichever came latest was defined as the date of CKD in post-natal follow-up<br><br>Kidney replacement therapy (KRT) was based on the procedure codes and billing codes for chronic dialysis (≥3 continuous months and ≥ 1 time in each month) and kidney transplantation.
Congenital anomalies of kidney and urinary tract (CAKUT)	**Renal agenesis, hypoplasia, or dysplasia**<br>ICD-9: 589, 7530, 75315; ICD-10: N270, N271, N279, Q600, Q601, Q602, Q603, Q604, Q605, Q606, Q614	
	**Cystic kidney disease**<br>ICD-9:7531, 75310, 75311, 75312, 75313, 75314, 75316, 75319; ICD-10: Q6100, Q6101, Q6102, Q6111, Q6119, Q612, Q613, Q615, Q618, Q619	
	**Other anomalies of kidney**<br>ICD-9: 753, 7533 (e.g., accessory kidney, giant kidney, ectopic kidney)<br>ICD-10: Q630, Q631, Q632, Q633, Q638, Q639	
	**Urinary tract dilation and anomalies of the urethra or ureters (UTD)**<br>ICD-9: 591, 596, 7532, 75320, 75321, 75322, 75329, 7534, 7535, 7536,7537,7538;<br>ICD-10: N131, N1330, N1339, N320, Q620, Q6210, Q6211, Q6212, Q6231, Q6232, Q6239, Q624, Q625, Q6260, Q6261, Q6262, Q6263, Q6269, Q627, Q628,<br>Q6410, Q6411, Q6412, Q6419, Q642,<br>Q6431, Q6432, Q6433, Q6439,<br>Q644, Q645, Q646, Q647, Q648, Q649	
	**Vesicoureteral-reflux with reflux nephropathy (VUR)**<br>ICD-9: 59371, 59372<br>ICD-10:N13721, N13722, N13731, N13732	
Non-CAKUT	**Glomerular disease:**<br>ICD-9: 580, 581,582,583,<br>ICD-10: N00, N01, N03, N04, N05, N08, N14, N159 (5838/5839)<br>***Systemic Lupus Erythematosus (SLE)***<br>ICD-9: 7100; ICD-10: M320, M3210, M3219, M328, M329<br>**Proteinuria**<br>ICD: 7910, ICD: N02 (not included: R310, R319, R311,R312)<br>**Other glomerular disease**<br>Diabetes with renal manifestations:<br>ICD-9: 2504, ICD-10: E1021, E1022, E1029, E1121, E1122, E1129, E1165<br>Renal artery aneurysm: ICD-9: 4421, ICD-10: I722<br>Goodpasture's syndrome/ Wegener granulomatosis:<br>ICD-9: 44621,4464, ICD-10: M310, M3119, M3130<br>Hemolytic-uremic syndrome: ICD-9: 28311, ICD-10:D593	

© 2024 American Medical Association. All rights reserved.

	Henoch-Schonlein purpura: ICD-9: 2870, ICD-10: N07<br>Rheumatic arthritis: ICD-9: 714, ICD-10:M05	
	**Chronic kidney diseases**<br>ICD-9: 585, 586, V451<br>ICD-10: N18, N19, Z992	
	**Other non-glomerular disease**<br>Hyperaldosteronism:<br>ICD-9: 2551, ICD-10:'E2601' 'E2602' 'E2609' 'E261' 'E2681' 'E2689' 'E269'<br>Atrophy of kidney: ICD-9: 587, ICD-10: N261, N269<br>Renal osteodystrophy: ICD-9: 588, 5881, 5888, 5889, ICD-10: N25 N250, N251, N2581, N2589, N259<br>Stone: ICD-9: 592, ICD-10: N200,<br>Hyperplasia of renal artery: ICD-9:4473, ICD-10: I7773<br>Bladder:<br>ICD-9: 59654, ICD-10: N310, N311, N319<br>Urinary obstruction: ICD9: 5996; ICD-10: N139	
	**Hypertensive, lipidoses, gout, liver related chronic kidney diseases**<br>Hypertension:<br>ICD-9: 4031, 40492, 40493, 40412, 40413; ICD-10: I129, I120, I1311, I132;	
Congenital anomalies by system		
Nerves	ICD-9: 740-742<br>ICD-10: Q000, Q001, Q002, Q050-Q059, Q0700-Q0703, Q01-Q04, Q06, Q07	≥1 ICD-9/10 codes at birth in out- or inpatient setting
Orofacial	ICD-9: 743, 744, 748.1, 749<br>ICD-10: Q100-Q107, Q110-Q113, Q120-Q124, Q128, Q129, Q130-Q135, Q1381, Q1389, Q139, Q140-Q143, Q148-Q150, Q158, Q159, Q16-Q18, Q30.1, Q30.2, Q30.8, Q35-Q37, Q385	
Heart/Circulatory	ICD-9: 745, 746, 747; ICD-10: Q20-Q24, Q25-Q28	
Digestive/Gastrointestinal	ICD-9: 750, 751; ICD-10: Q38-Q40, Q41-Q45	
Other Urinary (genital anomalies)	ICD-9: 752; ICD-10:Q54	
Musculoskeletal	ICD-9: 754, 755, 756; ICD-10: Q65-Q68, Q743, Q763, Q7641, Q7642, Q75-Q79	
Respiratory	ICD-9: 748 (excluded 748.1); ICD-10: Q30-Q34 (excluded Q30.1, Q30.2, Q30.8)	
Chromosomes	ICD-9: 758; ICD-10: Q554, Q90-Q99	
Skin/Dermatology	ICD-9: 757; ICD-10: N6482, Q80-Q84	
Others/Unspecifics	ICD-9: 759; ICD-10: E7871, E7872, Q85, Q87, Q89, Q992	

Variable	Code	Timing and Definition
**Maternal health (8)**		

© 2024 American Medical Association. All rights reserved.

Hypertension	ICD-9: 401-405<br>ICD-10: I10~I13,I15, N26.2	Pre-gestational period;<br>≥1 ICD-9/10 codes in out- or inpatient setting
Hyperlipidemia	ICD-9: 272;<br>ICD-10: E75.2, E75.3, E75.5, E75.6, E77, E78, E88.1, E88.2, E88.89	
Carlson comorbidity index [3]	**17 comorbid conditions:**<br>Acute myocardial infarction: ICD-9: 410,412,428; ICD-10: I21, I22, I252;<br>Congestive heart failure: ICD-9: 428; ICD-10: I50;<br>Peripheral vascular disease: ICD-9: 441, 4439, 7854, V434; ICD-10: I71, I790, I739, R02, Z958, Z959;<br>Cerebral vascular accident: ICD-9: 430–438; ICD-10: G45, G46, H340, I60, I61, I62, I63, I64, I65, I66, I67, I68, I69;<br>Dementia: ICD-9: 290; ICD-10: F00, F01, F02, F051;<br>Chronic pulmonary disease: ICD-9: 490–505; ICD-10: J40–J47, J60–J66;<br>Rheumatic disease: ICD-9: 7100, 7101, 7104, 7141,7142, 71481, 725; ICD-10: M05, M06, M32–M34, M351, M353;<br>Peptic ulcer disease: 531–534; ICD-10: K25–K28;<br>Mild liver disease/ moderate: ICd-9: 5712, 5714–5716; ICD-10: K702, K703, K717, K73, K74;<br>Moderate or severe liver disease: ICD-9: 5722, 5723, 5724, 5728; ICD-10: K729, K766, K767, K721;<br>Diabetes without complication: 2500–2503, 2507; ICD-10: E109, E119, E139, E149, E101, E111, E131, E141, E105, E115, E135, E145;<br>Diabetes with complication: 2504–2506; ICD-10: E102, E112, E132, E142 E103, E113, E133, E143 E104, E114, E134, E144;<br>Hemiplegia or paraplegia: ICD-9: 3441, 342; ICD-10: G81, G82;<br>Kidney disease: ICD-9: 582, 583, 585, 586, 588, V420, V451, V56; ICD-10: N01, N03, N052, N053, N054, N055, N056, N072, N073, N074, N18, N19, N25, Z490– Z492, Z940, Z992;<br>Any malignancy: ICD-9: 140–172, 174–1958, 200–208; ICD-10: C00–C26, C30–C34, C37– C41, C43, C45–C58, C60– C76, C80–C85, C88, C90–C96;<br>Metastatic solid tumor: ICD-9: 196–1991; ICD-10: C77–C80;<br>AIDS: 042–044; ICD-10: B20–B22, B24	
Gestational diabetes	ICD-9: 648.83; ICD10: O24	Gestational period;<br>≥1 ICD-9/10 codes in out- or inpatient setting
Gestational hypertension [4]	1.pre-existing HTN: ICD-9: 642.0x, 642.1x, 642.2x; ICD-10: O10<br>2.gestational HTN: ICD-9: 642.3x, 642.9x; ICD-10: O13.1, O13.2, O13.3, O13.9; O16.1, O16.2, O16.3, O16.9	
(Pre)eclampsia [4]	ICD-9: 642.5x, 642.6x, 642.7x ; ICD-10:O14, O15, O11	
Anemia [5]	anemia during pregnancy (ICD-9: 648.23; ICD-10: O99.0,), excluding hemorrhage: O99.1 (coagulation defects: O46.0, O67.0, O72.3)	
(Other) Gestational-related complications [6] (excluded	ICD-9: 640, 641, 642, 643, 644, 645, 646,647,648,649<br>ICD-10: O100-O104, O109, O111-O113, O119-O122, O131-O133, O139-O142, O149-O152, O159,	

© 2024 American Medical Association. All rights reserved.

gestational hypertension, hyperlipidemia, diabetes, preeclampsia and anemia)	O161-O163, O169, O200, O208-O212, O218, O219, O230-O235, O239-O241, O243, O244, O248, O249, O251-O253, O260-O264, O266-O269, O290-O296, O298, O299, O310, O330, O440, O441, O450, O458, O459, O460, O468, O469, O470, O471, O479-O481, O600-O602, O670, O678, O679, O861, O862, O905, O906, O908, O980-O998, O9A1-O9A5	
**Medication use [7]**	**Anatomy of Therapeutic Classification code**	Pre- and during gestational period; ≥1 prescription in out- or in-patient setting based on prescription records (medication name, dosage, date, number of pills and number of day's supply of the medication is to be taken)
Anti-microbial agents	J01GB	
Immunosuppressants	L01AA01 (cyclophosphamide), L04AA06 (mycophenolate), L04AD01 (cyclosporine A)	
Anti-hypertension	C02AB (methyldopa), C03CA01, C03CB01(Furosemide), C08CA05 (nifedipine), C09 (agents acting on renin-angiotensin system, excluded)	
Anti-hyperglycemia	A10BA02, A10BD (Metformin), Insulins (A10A), A10B, A10X (other drugs used in diabetes)	
Systemic glucocorticoids [2]	betamethasone, dexamethasone, methylprednisolone, prednisolone, triamcinolone, and hydrocortisone; excluded topical use of preparations	
Anti-diabetes	A10	
Statins	C10AA, C10BA,C10BX	
Antidepressants	N06A	
Anticonvulsants	N03	
Benzodiazepines	N05BA, N05CD, N05CF	

References:

1. 2016 Annual Report on Kidney Disease in Taiwan. National Health Research Institutes, Hsinchu, Taiwan. Available at https://lib.nhri.edu.tw/NewWeb/nhri/ebook/39000000431630.pdf. Accessed at March 1, 2023.
2. Tain YL, Li LG, Kuo HC and Hsu CN. Gestational Exposure to Maternal Systemic Glucocorticoids and Childhood Risk of CKD. Am J Kidney Dis. 2024 Mar 11: S0272-6386(24)00669-3. doi: 10.1053/j.ajkd.2024.01.523.
3. Quan H, Sundararajan V, Halfon P, et al. Coding algorithms for defining comorbidities in ICD-9-CM and ICD-10 administrative data. Medical care 2005:1130-9
4. Fingar KR, Mabry-Hernandez I, Ngo-Metzger Q, et al. Delivery Hospitalizations Involving Preeclampsia and Eclampsia, 2005–2014: Statistical Brief #222. 2017 Apr. In: Healthcare Cost and Utilization Project (HCUP) Statistical Briefs [Internet]. Rockville (MD): Agency for Healthcare Research and Quality (US); 2006 Feb-. Table 7, ICD-9-CM diagnosis codes defining preeclampsia/eclampsia and other types of hypertension complicating pregnancy, childbirth, and the puerperium. Available from: https://www.ncbi.nlm.nih.gov/books/NBK442039/table/sb222.t7/ (assessed 10/10/2020)
5. Sharma AJ, Ford ND, Bulkley JE, et al. Use of the Electronic Health Record to Assess Prevalence of Anemia and Iron Deficiency in Pregnancy. *The Journal of Nutrition*, 2021; 151: 3588–3595.
6. Centers for Disease Control and Prevention. How Does CDC Identify Severe Maternal Morbidity**?** Available from https://www.cdc.gov/reproductivehealth/maternalinfanthealth/smm/severe-morbidity-ICD.htm. (assessed 10/10/2022)
7. Schreuder MF, Bueters RR, Huigen MC, et al. Effect of drugs on renal development. *Clinical Journal of the American Society of Nephrology.* 2011;6(1):212-217.

© 2024 American Medical Association. All rights reserved.

eTable 3. Neonatal characteristics between comparison groups in the singleton-born child cohort (n=680,696)

Characteristics	Singleton-born children (n=680,696)	Gestational exposure (n=163,516)		Non-exposure (n=517,180)		*P* value
Boy sex, n (%)	357062 (52.46)	86224	(52.73)	270838	(52.37)	0.0105
Weight at birth, mean gm (±SD); kg, n (%)	3083(±436.13)	3065	(±452.93)	3088	(±430.53)	<.0001
<1	1623	535	(0.33)	1088	(0.21)	<.0001
1 to 1.5	2612	872	(0.53)	1740	(0.34)	
1.5 to 2.5	41073	10855	(6.64)	30218	(5.84)	
2.5-4	625362	148867	(91.04)	476495	(92.13)	
>4	10026	2387	(1.46)	7639	(1.48)	
Gestational age, mean (SD), week; n (%)	38(±1.60)	38	(±1.70)	38	(±1.57)	<.0001
<27	1133	361	(0.22)	772	(0.15)	<.0001
27 to 34	7881	2433	(1.49)	5448	(1.05)	.
34 to 36	39458	10707	(6.55)	28751	(5.56)	.
37 to 40	612278	145452	(88.95)	466826	(90.26)	.
>40	19946	4563	(2.79)	15383	(2.97)	
SGA/AGA/LGA, n (%)						
SGA	63319	15545	(9.51)	47774	(9.24)	0.0021
AGA	571507	136864	(83.70)	434643	(84.04)	
LGA	45870	11107	(6.79)	34763	(6.72)	
First-min Apgar score, n (%)						<.0001
≤7	51387	13894	(8.50)	37493	(7.25)	
>7	628773	149509	(91.43)	479264	(92.67)	
Unknown	536	113	(0.07)	423	(0.08)	
Fifth-min Apgar score, n (%)						<.0001
≤7	9357	2710	(1.66)	6647	(1.29)	
>7	670827	160701	(98.28)	510126	(98.64)	.
Unknown	512	105	(0.06)	407	(0.08)	

P value: independent t test for numeric data (mean, standard deviation, SD); and chi-square test for n (%) data difference between gestational and non-exposure group; SGA, small gestational age; AGA, average gestational age; LGA, large gestational age

© 2024 American Medical Association. All rights reserved.

eTable 4 Uses of NSAIDs by trimester in pregnancy in the singleton-born child cohort (n=163,516)

Exposure	Trimester (n=163516)			Gestational week (n=163516)	
	First	Second	Third	≤20 weeks	>20 weeks
**NSAIDs,** n (%)	99846 (61.06)	54034 (33.05)	43447 (26.57)	124558 (76.17)	61403 (37.55)
Aspirin (ASA)	5091 (5.10)	4541 (8.40)	3397 (7.82)	7782 (6.25)	4785 (7.79)
Non-ASA NSAIDs	95721 (95.87)	49847 (92.25)	40251 (92.64)	118417 (95.07)	57720 (94.00)
**Lower COX- 2 selectivity,** n (%)	**36877 (36.93)**	**16108 (29.81)**	**12986 (29.89)**	**45108 (36.21)**	**19211 (31.29)**
Indomethacin	1049 (1.05)	3749 (6.94)	3051 (7.02)	2960 (2.38)	4785 (7.79)
Ibuprofen	22309 (22.34)	6832 (12.64)	4843 (11.15)	25920 (20.81)	7403 (12.06)
Naproxen	3999 (4.01)	1868 (3.46)	1463 (3.37)	5095 (4.09)	2127 (3.46)
Acemetacin	1919 (1.92)	210 (0.39)	213 (0.49)	2043 (1.64)	291 (0.47)
Ketorolac	4412 (4.42)	1805 (3.34)	1947 (4.48)	5419 (4.35)	2664 (4.34)
Aceclofenac	713 (0.71)	78 (0.14)	119 (0.27)	761 (0.61)	147 (0.24)
Ketoprofen	2098 (2.10)	1162 (2.15)	979 (2.25)	2764 (2.22)	1396 (2.27)
Tolmetin (sodium)	34 (0.03)	5 (0.01)	≦3	35 (0.03)	7 (0.01)
Fenoprofen (calcium)	40 (0.04)	9 (0.02)	≦3	46 (0.04)	4 (0.01)
Piroxicam	1400 (1.40)	431 (0.80)	355 (0.82)	1637 (1.31)	514 (0.84)
Tenoxicam	296 (0.30)	76 (0.14)	129 (0.30)	338 (0.27)	161 (0.26)
Meclofenamic acid	567 (0.57)	385 (0.71)	324 (0.75)	807 (0.65)	461 (0.75)
Nabumetone	53 (0.05)	5 (0.01)	4 (0.01)	58 (0.05)	5 (0.01)
**Higher COX-2 selectivity,** n (%)	**67913 (68.02)**	**35973 (66.57)**	**29001 (66.75)**	**85358 (68.53)**	**41503 (67.59)**
Mefenamic acid	30440 (30.49)	16794 (31.08)	14486 (33.34)	39357 (31.60)	20424 (33.26)
Diclofenac	40530 (40.59)	19995 (37.00)	15090 (34.73)	50652 (40.67)	22284 (36.29)
Alclofenac	8 (0.01)	0	0	8 (0.01)	0
meloxicam	342 (0.34)	110 (0.20)	97 (0.22)	406 (0.33)	126 (0.21)
Tolfenamic acid	44 (0.04)	23 (0.04)	18 (0.04)	52 (0.04)	23 (0.04)
Flufenamic acid	110 (0.11)	23 (0.04)	33 (0.08)	123 (0.10)	41 (0.07)
Celecoxib	54 (0.05)	20 (0.04)	10 (0.02)	63 (0.05)	15 (0.02)
Etoricoxib	45 (0.05)	≦3	5 (0.01)	47 (0.04)	6 (0.01)
Niflumic acid	18 (0.02)	4 (0.01)	0	19 (0.02)	≦3
Nimesulide	140 (0.14)	101 (0.19)	67 (0.15)	200 (0.16)	105 (0.17)
Etodolac micronized	226 (0.23)	172 (0.32)	137 (0.32)	312 (0.25)	200 (0.33)
Fenbufen	271 (0.27)	59 (0.11)	41 (0.09)	308 (0.25)	61 (0.10)
**Uncertain,** n (%)	**704 (0.71)**	**394 (0.73)**	**299 (0.69)**	**928 (0.75)**	**418 (0.68)**
Benzydamine HCl	165 (0.17)	54 (0.10)	30 (0.07)	190 (0.15)	55 (0.09)
Mepirizole	543 (0.54)	340 (0.63)	269 (0.62)	742 (0.60)	363 (0.59)

1st trimester: last menstrual period to <13 weeks; 2nd trimester: 13-26 weeks; 3rd trimester: ≥27 weeks to delivery; Number for event rate ≤3 is not shown due to the data security requirement

© 2024 American Medical Association. All rights reserved.

eTable 5. Incidence rate of study outcome in the singleton-born child cohort (n=680,696)

Study outcome	Singleton-born children (n=680,696)			Gestational exposure (n=163,516)			Non-exposure (n=517,180)			wHR	*95% CI*	*P value*
	person-year	n (%)	n/1000PY	person-year	n (%)	n/1000 PY	person-year	n (%)	n/1000 PY			
CKD	6381992.54	10547 (1.55)	1.65	1562367.99	2792 (1.71)	1.79	4819624.55	7755 (1.50)	1.61	1.10	(1.05-1.15)	0.0001
CAKUT	6429047.48	4981 (0.73)	0.77	1574969.42	1311 (0.80)	0.83	4854078.05	3670 (0.71)	0.76	1.08	(1.01-1.16)	0.0233
Non-CAKUT	6409892.42	7144 (1.05)	1.11	1569703.34	1913 (1.17)	1.22	4840189.08	5231 (1.01)	1.08	1.12	(1.06-1.19)	<0.001
KRT	6469940.26	273 (0.04)	0.04	1585943.71	68 (0.04)	0.04	4883996.55	205 (0.04)	0.04	NA		
All-cause mortality	6470908.19	2533 (0.37)	0.39	1586166.25	734 (0.45)	0.46	4884741.93	1799 (0.35)	0.37	1.12	(1.03-1.23)	0.0110

CKD, chronic kidney disease; CAKUT, congenital anomalies of kidney and urinary tract; KRT, kidney replacement therapy, including chronic dialysis and kidney transplantation; PY, person-year;

wHR: hazard ratio derived from the stabilized inverse probability of treatment weighted Cox regression model with robust sandwich estimator, adjustment with fetal characteristics in the singleton-born cohort (n=680,696).

© 2024 American Medical Association. All rights reserved.

eTable 6. In sensitivity analyses, study outcomes in the additional 60-day exposure to NSAIDs period after the date of birth (n=680,696)

	Gestational prolonged exposure (n=521,577)	Non-exposure (n=159,119)	wHR	95% CI	*p-value*
Any CKD	8155 (1.56)	2392 (1.50)	1.01	(0.96 -1.06)	0.7563
CAKUT	3824 (0.73)	1157 (0.73)	0.97	(0.91 -1.04)	0.3624
Non-CAKUT	5547 (1.06)	1597 (1.00)	1.03	(0.97 -1.09)	0.2982
KRT	222 (0.04)	51 (0.03)	1.26	(0.92 -1.73)	0.1447
All-cause mortality	2026 (0.39)	507 (0.32)	1.18	(1.06 -1.31)	0.0018

CKD, chronic kidney disease; CAKUT, congenital anomalies of kidney and urinary tract; KRT, kidney replacement therapy, including chronic dialysis and kidney transplantation; wHR: conditional hazard ratio derived from the stabilized IPTW Cox proportional progression model with robust sandwich estimator, adjustment with offspring characteristics.

The number of newborns who were actually treated with NSAIDs in 60 days after birth is uncertain because the newborns typically receive an unique individual identifier and enroll in Taiwan national health program within 30-60 days after the birth. Using maternal prescription records as proxies may be one way to explore the concern of NSAID nephrotoxicity in newborns, though the timing of exposure differs from that in fetuses.

eTable 7. Dose response between individual NSAID and risk of CKD in the singleton-born child cohort

Exposure	No of children	No of children with event	%	wHR	95% CI	*p-value*
**Aspirin, cumulative dose, mg**						
<5000	6061	89	(1.47)	1.00		
5000-7500	663	11	(1.66)	1.47	(0.72 -3.02)	0.2925
>7500	2308	35	(1.52)	0.86	(0.55 -1.33)	0.4882
**Naproxen , cumulative dose, mg**						
<3000	4181	71	(1.70)	1.00		
3000-4000	1064	23	(2.16)	1.43	(0.85 -2.40)	0.1785
>4000	1732	28	(1.62)	1.08	(0.66 -1.76)	0.7603

wHR: conditional hazard ratio derived from the stabilized IPTW Cox proportional progression model with robust sandwich estimator, adjustment with offspring characteristics.

© 2024 American Medical Association. All rights reserved.

eTable S8. Neonatal characteristics in the sibling-matched child cohort (n=344,559)

Characteristics	**Sibling-matched children**	**Gestational exposure (n=68,275)**	**Non-exposure (n=276,284)**	*p-value*
**Sex, n (%)**				
boys	175523	34873 (51.08)	140650 (50.91)	0.4291
girls	169036	33402 (48.92)	135634 (49.09)	
**Birth weight (gm), mean (SD)**	3091(423.41)	3072.21 (440.62)	3095.34 (418.92)	<.0001
<1000	756	213 (0.31)	543 (0.20)	<.0001
1000 to <1500	1087	297.00 (0.44)	790.00 (0.29)	
1500 to <2500	18755	4235.00 (6.20)	14520.00 (5.26)	
2500 to ≤4000	319353	62585 (91.67)	256768 (92.94)	
>4000	4608	945 (1.38)	3663 (1.33)	
**Gestational age (week), mean (SD)**	38(1.56)	38.29 (1.66)	38.40 (1.54)	<.0001
21-27	600	153 (0.22)	447 (0.16)	<.0001
27-34	3517	898 (1.32)	2619 (0.95)	
34-37	18315	4129.00 (6.05)	14186.00 (5.13)	
37-40	312619	61319 (89.81)	251300 (90.96)	
>40	9508	1776 (2.60)	7732 (2.80)	
**SGA/AGA/LGA, n (%)**				
SGA	28964	5996 (8.78)	22968 (8.31)	<.0001
AGA	292925	57704 (84.52)	235221 (85.14)	
LGA	22670	4575.00 (6.70)	18095.00 (6.55)	
**First-min Apgar score, n (%)**				<.0001
<=7	22548	5138 (7.53)	17410 (6.30)	
>7	321586	63074 (92.38)	258512 (93.57)	
Unknown	425	63 (0.09)	362 (0.13)	
**Fifth-min Apgar score, n (%)**				<.0001
<=7	4210	1005 (1.47)	3205 (1.16)	
>7	339950	67210 (98.44)	272740 (98.72)	
Unknown	399	60 (0.09)	339 (0.12)	

P value: independent t test for numeric data (mean, standard deviation, SD); and chi-square test for n (%) data difference between gestational and non-exposure group; SGA, small gestational age; AGA, average gestational age; LGA, large gestational age

© 2024 American Medical Association. All rights reserved.

eTable 9. Incidence rate of study outcome in the sibling-matched child cohort (n=344,559)

Study outcome	Sibling-matched children (n=344,559)			Gestational exposure (n=68,275)			Non-exposure (n=276,284)		
	person-year	n (%)	n/1000 PY	person-year	n (%)	n/1000 PT	person-year	n (%)	n/1000 PY
Any CKD	3197427.52	4913 (1.43)	1.54	641193.58	1071 (1.57)	1.67	2556233.94	3842 (1.39)	1.50
CAKUT	3220386.96	2182 (0.63)	0.68	646340.41	461 (0.68)	0.71	2574046.55	1721 (0.62)	0.67
Non-CAKUT	3209642.18	3418 (0.99)	1.06	643900.13	745 (1.09)	1.16	2565742.04	2673 (0.97)	1.04
KRT	3229700.20	155 (0.04)	0.05	647444.44	49 (0.07)	0.08	2582255.76	106 (0.04)	0.04
All-cause mortality	3238548.37	2050 (0.59)	0.63	650240.84	490 (0.72)	0.75	2588307.53	1560 (0.56)	0.60

CKD, chronic kidney disease; CAKUT, congenital anomalies of kidney and urinary tract; KRT, kidney replacement therapy, including chronic dialysis and kidney transplantation; PY, person-year

© 2024 American Medical Association. All rights reserved.

eTable 10. Associations of study outcomes with gestational exposure to NSAIDs in the sibling-matched child cohort analyses (n=344,559)

	Sibling number.	Event number (%)	Stabilized IPTW Cox model			Stratified PS Cox model		
			wHR	95% CI	*p-value*	sHR	95% CI	*p-value*
**Primary analysis**	344559	4913 (1.43)	1.05	(0.97 -1.13)	0.2124	1.05	(0.98 -1.13)	0.1446
**Exposure timing during pregnancy**								
1st trimester	41357	651 (1.57)	1.06	(0.97 -1.16)	0.2122	1.06	(0.97 -1.15)	0.1865
2nd trimester	20948	326 (1.56)	1.07	(0.94 -1.21)	0.3286	1.04	(0.93 -1.17)	0.4982
3rd trimester	17005	269 (1.58)	1.07	(0.93 -1.23)	0.3226	1.05	(0.93 -1.19)	0.4375
≤20 gestational weeks	51408	805 (1.57)	1.06	(0.97 -1.15)	0.2006	1.06	(0.98 -1.14)	0.1834
>20 gestational weeks	24180	386 (1.60)	1.07	(0.96 -1.21)	0.2387	1.06	(0.95 -1.18)	0.2801
**Sex**								
Boys	175523	2612 (1.49)	1.01	(0.91 -1.12)	0.9080	1.01	(0.92 -1.12)	0.7826
Girls	169036	2301 (1.36)	1.10	(0.99 -1.22)	0.0841	1.10	(1.00 -1.22)	0.0571
**Birthweight, gm**								
<2500	20598	303 (1.47)	0.97	(0.72 -1.30)	0.8457	0.98	(0.75 -1.28)	0.8798
≥2500	323961	4610 (1.42)	1.05	(0.98 -1.14)	0.1757	1.06	(0.99 -1.14)	0.1167
**Gestational age, week**								
<37	22432	380 (1.69)	1.00	(0.77 -1.30)	0.9881	0.97	(0.77 -1.24)	0.8251
≥37	322127	4533 (1.41)	1.05	(0.97 -1.14)	0.1916	1.06	(0.99 -1.14)	0.1074

1st trimester: last menstrual period to <13 weeks; 2nd trimester: 13-26 weeks; 3rd trimester: ≥27 weeks to delivery

wHR: conditional hazard ratio derived from the stabilized inverse probability of treatment (IPTW) weighted Cox regression model; sHR. hazard ratio derived from the stratified propensity score (PS) Cox regression model (5 strata), and both models adjusted with the same offspring characteristics using robust sandwich estimator in the sibling-matched cohort (n=344,559)

© 2024 American Medical Association. All rights reserved.

eTable S11. Descriptions of the common NSAIDs used in the study

NSAIDs	Category	Pregnancy warning for fetal health	Placenta transferability	Pharmacokinetics
**Acetic acid derivatives**				
Indomethacin (active acemetacin metabolite)	Fetal harm has been demonstrated [5]	1. Avoid use in pregnant women between about 20 to 30 weeks due to oligohydramnios/fetal kidney dysfunction, and 30 weeks later due to premature closure of the fetal ductus arteriosus [1]<br>2. Persistent pulmonary hypertension of the newborn may occur if used in the 3rd trimester [8].<br>3. Indomethacin use during the latter part of pregnancy may cause severe fetal toxicity. Short-term use, such as 24–48 hours, lessens the risk [2-4]	Mean ratio of maternal/fetal indomethacin levels is 0.97 [6].<br>The Fetal/maternal Cmax ratio: 0.68 and 1.01, the Fetal/maternal AUC 0-6h ratio: 0.69 and 1.02 during second trimester and third trimester, respectively [7].<br>Mean amniotic fluid level was significantly lower than the maternal and fetal concentrations [6].	T1/2 : 5-10 hrs<br>Tmaxs: Oral:1.3 to 2 hrs<br>Oral BA/BE: 100%<br>Protein binding: 99%<br>Kidney excretion: 60%
Diclofenac	Fetal harm has been demonstrated [5]	1. Avoid use in pregnant women between about 20 to 30 weeks due to oligohydramnios/fetal renal dysfunction, and 30 weeks later due to premature closure of the fetal ductus arteriosus [1]<br>2. Women attempting to conceive should not use ibuprofen, due to block blastocyst implantation in animal models [9]	Mean maternal serum/fetal tissue ratio: 0.95. (range 0.05-4.26) [12]	T1/2:1-2 hrs;<br>Tmax, Oral: 1 hr<br>Oral BA/BE: 50%<br>Protein binding: 99%<br>Kidney excretion: 65%
Acemetacin	Fetal harm has been demonstrated [5]	1. Avoid use in pregnant women between about 20 to 30 weeks due to oligohydramnios/fetal renal dysfunction, and 30 weeks later due to premature closure of the fetal ductus arteriosus [1]	The molecular weight (about 416)	T1/2: 4.5hrs;<br>Tmax: oral 2.5hrs<br>Protein binding: 90%<br>Kidney excretion: 40%
Ketorolac	Fetal harm has been demonstrated [5]	1. Avoid use in pregnant women between about 20 to 30 weeks due to	The molecular weight (about 376) is Low enough that passage to the	T1/2:5-6 hrs;<br>Oral BA/BE: 80-100%

© 2024 American Medical Association. All rights reserved.

		oligohydramnios/fetal kidney dysfunction, and 30 weeks later due to premature closure of the fetal ductus arteriosus [1]<br>2. ketorolac was contraindicated in labor and delivery because its prostaglandin synthesis inhibitory effect may adversely affect fetal circulation and inhibit uterine contractions [13]<br>3. Consider withdrawal of ketorolac in women who are having difficulties conceiving or undergoing investigation of infertility [14]	embryo and fetus.	Tmax: 20-60 mins<br>Protein binding> 99%<br>Kidney excretion: mainly
Aceclofenac	Contraindicated [5]	1. Aceclofenac is contraindicated during the third trimester of pregnancy. Do not administer aceclofenac during the first or second trimester of pregnancy unless clearly necessary, administering as low a dose as possible for the shortest duration as possible [15]	Low molecular weight of 354.19	T1/2: 4hrs<br>Tmax: 1.25-3hrs<br>Protein binding> 99%<br>Kidney excretion: 70-80%
**Oxicams**				
Meloxicam	Fetal harm has been demonstrated [5]	1. Avoid use in pregnant women between about 20 to 30 weeks due to oligohydramnios/fetal kidney dysfunction, and 30 weeks later due to premature closure of the fetal ductus arteriosus [1]	The molecular weight (about 351) is low enough that transfer to the fetus. [16]	T1/2: 20hrs<br>Tmax: 5-6hrs<br>Oral BA/BE: 89%<br>Protein binding> 99%<br>Kidney excretion: metabolites <0.25%
**Propionic acid derivatives**				
Naproxen	Fetal harm has been demonstrated [5]	1. Avoid use in pregnant women between about 20 to 30 weeks due to oligohydramnios/fetal renal dysfunction, and 30 weeks later due to premature closure of the fetal ductus arteriosus [1]<br>2. Persistent pulmonary hypertension of the newborn may occur if used in the 3rd	Low molecular weight (about 230), naproxen readily crosses the placenta to the fetus.<br>Mean fetal/maternal drug ratio:0.092. (range 0.022-0.155) [10]	T1/2:12-17hrs<br>Tmax: 3-5hrs<br>Oral BA/BE: %<br>Protein binding> 99%<br>Kidney excretion: 66-92%

© 2024 American Medical Association. All rights reserved.

		trimester [8].		
Ibuprofen		1.Avoid use in pregnant women between about 20 to 30 weeks due to oligohydramnios/fetal renal dysfunction, and 30 weeks later due to premature closure of the fetal ductus arteriosus [1]<br>2.Persistent pulmonary hypertension of the newborn may occur if used in the 3rd trimester [8].<br>3.Women attempting to conceive should not use ibuprofen, due to block blastocyst implantation in animal models [9]	The molecular weight (about 206) suggests that the drug will cross the placenta throughout pregnancy	T1/2: 1.8-2 hrs;<br>Tmax, Oral: 1 to 2 hrs<br>Oral BA/BE: 92%<br>Protein binding: 99%<br>Kidney excretion: 45% to 79% as metabolites
Ketoprofen		1.Avoid use in pregnant women between about 20 to 30 weeks due to oligohydramnios/fetal renal dysfunction, and 30 weeks later due to premature closure of the fetal ductus arteriosus [1]<br>2. Persistent pulmonary hypertension of the newborn may occur if used in the 3rd trimester [8].	Low molecular weight (about 254), ketoprofen crosses the human placenta to the fetus and is detectable in the newborn. [17]	T1/2: 1.1-4 hrs;<br>Tmax, Oral: 0.5 to 2 hrs<br>Oral BA/BE: 100%<br>Protein binding: 99%<br>Kidney excretion: 80%
**Fenamates**				
Mefenamic acid	Fetal harm has been demonstrated [5]	1. Avoid use in pregnant women between about 20 to 30 weeks due to oligohydramnios/fetal kidney dysfunction, and 30 weeks later due to premature closure of the fetal ductus arteriosus [1]<br>2. NSAID use is not recommended in women attempting to conceive as it may impair female fertility, due to block blastocyst implantation in animal models [9]	Low molecular weight (about 241), mefenamic acid crosses the human placenta to the fetus.<br><br>Fetal concentrations of the drug were 32%–54% of the maternal plasma concentrations [11].	T1/2:2hrs;<br>Tmax, Oral: 2 to 4 hrs<br>BA/BE: 100%<br>Protein binding: 90%<br>Kidney excretion: 52%
Meclofenamic acid			Low molecular weight (<500)	T1/2: 1-5hrs<br>Tmax, Oral: 0.9 hrs<br>BA/BE: 100%

© 2024 American Medical Association. All rights reserved.

				Protein binding: 99%<br>Renal excretion: 70% as metabolites
**Coxibs**				
Celecoxib	Fetal harm has been demonstrated [5]	1. Avoid use in pregnant women between about 20 to 30 weeks due to oligohydramnios/fetal renal dysfunction, and 30 weeks later due to premature closure of the fetal ductus arteriosus [1]<br>2. Consider withdrawal celecoxib in women who have difficulties conceiving or who are undergoing infertility investigation [18].	Molecular weight (about 381) is low enough that passage to the embryo–fetus. [19]	T1/2: 11hrs<br>Tmax, Oral: rapidly<br>BA/BE: 100%<br>Protein binding: 97%<br>Renal excretion: 27% as metabolites
Parecoxib			Low molecular weight (370.4)	Injection T1/2: 22 mins to 8 hrs (metabolite)<br>Protein binding: 98%
Etoricoxib			Low molecular weight (359)	T1/2: 22hrs<br>Tmax, Oral: rapidly<br>BA/BE: 100%<br>Protein binding: 92%
**Anti-platelet aggregation**				
Aspirin	Fetal harm has been demonstrated [5]	1. Persistent pulmonary hypertension of the newborn may occur if used in the 3rd trimester [8].<br>2. Evidence has demonstrated fetal abnormalities or risks when used during pregnancy. Prescribe an alternative, if possible. Advise women of childbearing potential of possible fetal risk.	Low molecular weight (180)	T1/2: 3.5-4.5hrs<br>Tmax, Oral: rapidly<br>BA/BE: 100%<br>Protein binding: 50-90%<br>Kidney excretion: mainly

References

1. US Food & Drug Administration (FDA): FDA recommends avoiding use of NSAIDs in pregnancy at 20 weeks or later because they can

© 2024 American Medical Association. All rights reserved.

result in low amniotic fluid. US Food & Drug Administration (FDA). Silver Spring, MD. 2020. As accessed 2020-10-19.

2. Besinger RE, Niebyl JR, Keyes WG, Johnson TRB. Randomized comparative trial of indomethacin and ritodrine for the long-term treatment of preterm labor. *Am J Obstet Gynecol* 1991;164:981–8.
3. Abou-Ghannam G, Usta IM, Nassar AH. Indomethacin in pregnancy: applications and safety. *Am J Perinatol*. 2012;29:175-86. doi: 10.1055/s-0031-1284227.
4. Haram K, Mortensen JH, Morrison JC. Tocolysis for acute preterm labor: does anything work. *J Matern Fetal Neonatal Med*. 2015;28:371-8. doi: 10.3109/14767058.2014.918095.
5. Merative™ Micromedex® DRUGDEX® (electronic version). Merative, Ann Arbor, Michigan, USA. Available at: https://www.micromedexsolutions.com/ (cited: *December 12 ,2023)*
6. Moise KJ Jr, Ou C-N, Kirshon B, Cano LE, Rognerud C, Carpenter RJ Jr. Placental transfer of indomethacin in the human pregnancy. *Am J Obstet Gynecol* 1990;162:549–54.
7. Pillai VC, Shah M, Rytting E, Nanovskaya TN, Wang X, Clark SM, Ahmed MS, Hankins GDV, Caritis SN, Venkataramanan R. Prediction of maternal and fetal pharmacokinetics of indomethacin in pregnancy. *Br J Clin Pharmacol*. 2022;88:271-281. doi: 10.1111/bcp.14960.
8. Alano MA, Ngougmna E, Ostrea EM Jr, Konduri GG. Analysis of nonsteroidal antiinflammatory drugs in meconium and its relation to persistent pulmonary hypertension of the newborn. *Pediatrics* 2001;107:519–23. (ibuprofen, naproxen, indomethacin, and aspirin)
9. Matt DW, Borzelleca JF. Toxic effects on the female reproductive system during pregnancy, parturition, and lactation. In: WitorschRJ, ed. Reproductive Toxicology. 2nd ed. New York, NY: Raven Press, 1995:175–93.
10. Siu SSN, Yeung JHK, Lau TK. An in-vivo study on placental transfer of naproxen in early human pregnancy. *Hum Reprod* 2002;17:1056–9.
11. MacKenzie IZ, Graf AK, Mitchell MD. Prostaglandins in the fetal circulation following maternal ingestion of a prostaglandin synthetase inhibitor during mid-pregnancy. *Int J Gynaecol Obstet* 1985;23:455–8.
12. Siu SSN, Yeung JHK, Lau TK. A study on placental transfer of diclofenac in first trimester of human pregnancy. *Hum Reprod* 2000;15:2423–5.

© 2024 American Medical Association. All rights reserved.

13. Reinhart DI. Minimising the adverse effects of ketorolac. Drug Saf. 2000 Jun;22(6):487-97. doi: 10.2165/00002018-200022060-00007.
14. Product Information: SPRIX(R) nasal spray, ketorolac tromethamine nasal spray. Egalet US Inc. (per FDA), Wayne, PA, 2016.
15. Product Information: Aceclofenac oral tablets, aceclofenac oral tablets. Accord Healthcare Limited (per MHRA), Middlesex, United Kingdom, 2015.
16. Levin DL. Effects of inhibition of prostaglandin synthesis on fetal development, oxygenation, and the fetal circulation. *Semin Perinatol* 1980;4:35–44.
17. Labat L, Llanas B, Demotes-Mainard F, Lagrange F, Demarquez JL, Bannwarth B. Accumulation of S-ketoprofen in neonates after maternal administration of the racemate. *Fundam Clin Pharmacol* 1995;9:62. As cited in Lagrange F, Pehourcq F, Bannwarth B, Leng JJ, Saux MC. Passage of S-(+)- and R-(−)-ketoprofen across the human isolated perfused placenta. *Fundam Clin Pharmacol* 1998;12:286–91.
18. Product Information: ELYXYB oral solution, celecoxib oral solution. Dr. Reddy's Laboratories Limited (per FDA), Charlotte, NC, 2020.
19. Product information. Celebrex. G.D. Searle LLC Division of Pfizer, 2019.

In summary, evidence has demonstrated fetal abnormalities or risks when NSAIDs used during pregnancy. NSAIDs use in pregnancy < 20 weeks gestation should be based on a benefit-risk assessment. If NSAID use is necessary between 20- and 30-weeks' gestation, limit use to the lowest effective dose for the shortest duration possible; ultrasound monitoring of amniotic fluid should be considered if NSAIDs use extends beyond 48 hours; if oligohydramnios occurs, discontinue NSAID and treat appropriately; NSAID use is not recommended in women attempting to conceive as it may impair female fertility.

© 2024 American Medical Association. All rights reserved.

eFigure 1. Study design, exposure and outcome time frame

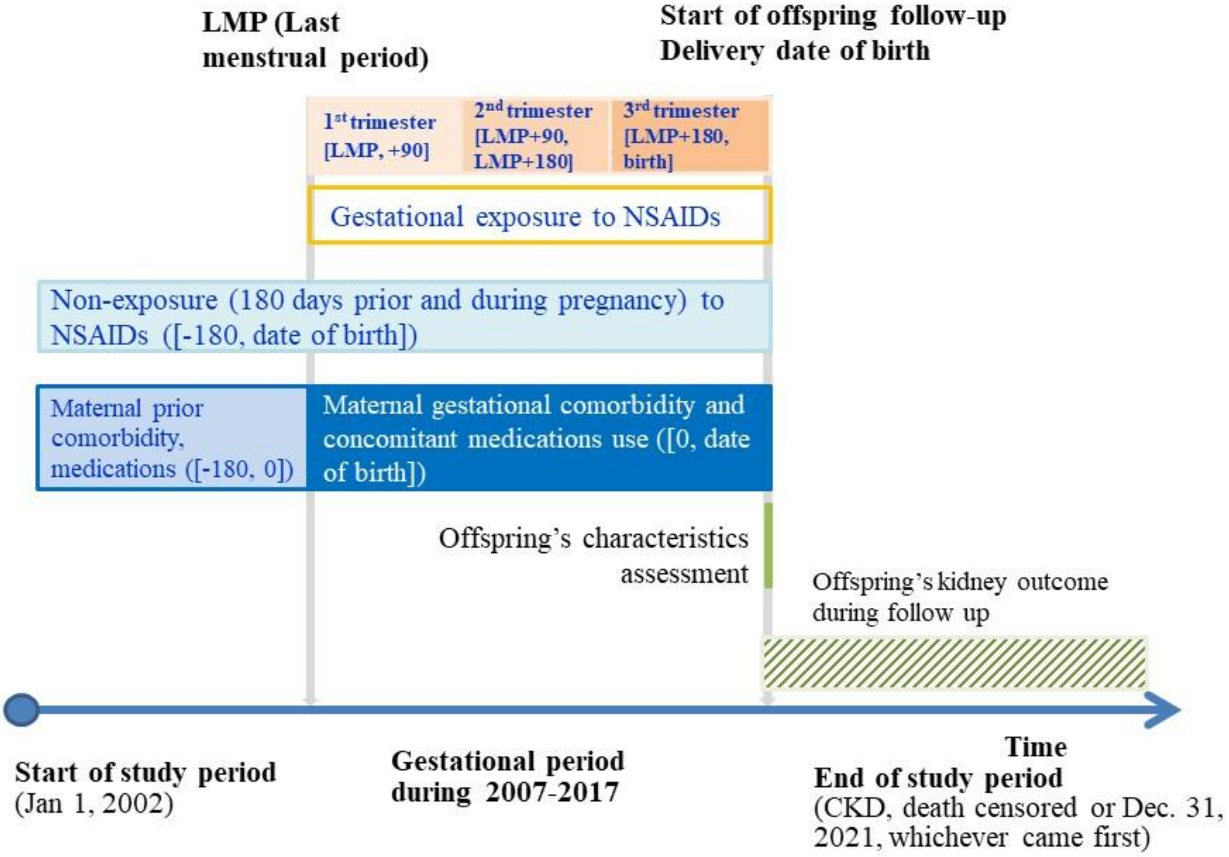

© 2024 American Medical Association. All rights reserved.

eFigure 2. The distributions of logit of the propensity score of subjects between gestational exposure and non-exposure to NSAIDs groups

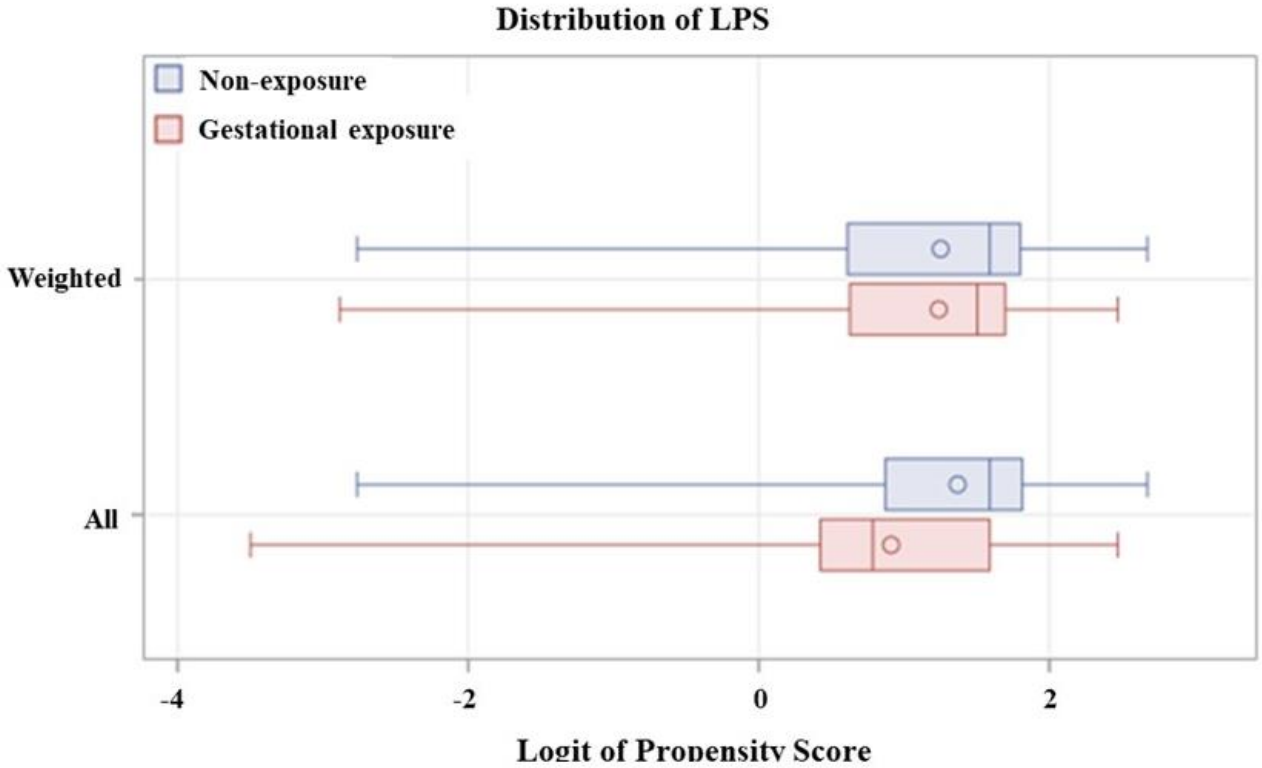

© 2024 American Medical Association. All rights reserved.

eFigure 3. Cumulative incidence of CKD in offspring over 15 years' follow-up

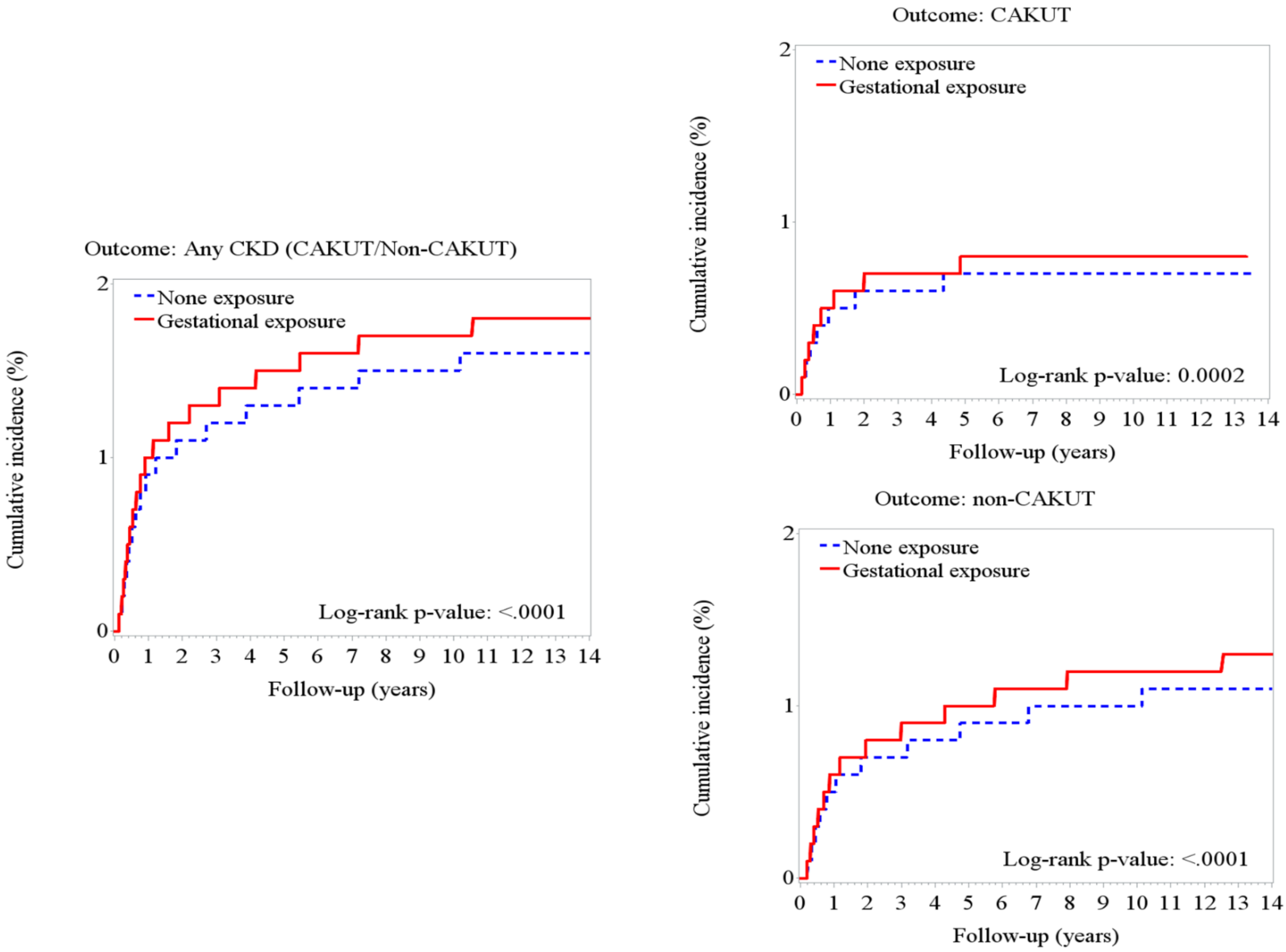

© 2024 American Medical Association. All rights reserved.
