# Supplementary material for: Gestational Exposure to Nonsteroidal Anti-Inflammatory Drugs and Risk of Chronic Kidney Disease in Childhood
Source: JAMA Pediatr. 2024 Dec 23;179(2):171–8. doi: 10.1001/jamapediatrics.2024.4409 (PMC11791701; doi:10.1001/jamapediatrics.2024.4409)
Supplement: Supplement 2. — Data Sharing Statement [file jamapediatr-e244409-s002.pdf]

Data Sharing Statement

Tain. Gestational Exposure to Nonsteroidal Anti-Inflammatory Drugs and Risk of Chronic Kidney Disease in Childhood. *JAMA Pediatr*. Published December 23, 2024. doi:10.1001/jamapediatrics.2024.4409

Data
Data available: Yes
Data types: Deidentified participant data, Other (please specify)
Additional Information: Data availability: no
How to access data: Data availability: no
When available: With publication

Supporting Documents
Document types: None

Additional Information
Who can access the data: corresponding author
Types of analyses: none
Mechanisms of data availability: none
